# Clinicopathological Features of Alopecia With an Emphasis on Etiology and Histopathological Characteristics of Scarring Alopecia

**DOI:** 10.7759/cureus.27661

**Published:** 2022-08-03

**Authors:** Atif A Hashmi, Khushbakht Rashid, Rubia Ali, Tanim Ud Dowlah, Abrahim H Ali, Muhammad Asad Diwan, Umair Arshad Malik, Muhammad Irfan, Shamail Zia, Adeel Ahmad

**Affiliations:** 1 Pathology, Liaquat National Hospital and Medical College, Karachi, PAK; 2 Internal Medicine, Liaquat National Hospital and Medical College, Karachi, PAK; 3 Internal Medicine, Bangladesh Medical College, Dhaka, BGD; 4 Pathology, Aga Khan University, Karachi, PAK; 5 Internal Medicine, Aga Khan University, Karachi, PAK; 6 Statistics, Liaquat National Hospital and Medical College, Karachi, PAK; 7 Pathology, Ziauddin University, Karachi, PAK; 8 Pathology, Roswell Park Comprehensive Cancer Center, Buffalo, USA

**Keywords:** lichen planopilaris (lpp), pseudopelade of brocq, discoid lupus erythematosus, non-scarring alopecia, scarring alopecia

## Abstract

Introduction

Alopecia is a common dermatological condition with varied etiologies based on age, gender and geographic location. Non-cicatricial (non-scarring) alopecia is more common but often not biopsied. Alternatively, primary cicatricial (scarring) alopecia is diagnostically more challenging and more commonly biopsied to determine the etiology. In this study, we evaluated the clinicopathological characteristics of alopecia in our population.

Methods

We conducted a retrospective study at the Department of Histopathology, Liaquat National Hospital and Medical College, Pakistan. A total of 104 patients were enrolled in the study who underwent scalp biopsy for alopecia over a period of 11 years. Clinical data were obtained from clinical referral forms, which included age, sex of the patients and duration of the lesions. Three hematoxylin and eosin-stained tissue sections, along with periodic acid-Schiff (PAS), PAS with diastase and collagen stains were examined, and histopathological diagnosis was rendered.

Results

Our study demonstrated that alopecia was more prevalent among females, accounting for 73.1% of cases. Most of the patients belonged to the age group of <35 years (53.8%). The type of alopecia in 88.5% of the cases was scarring, while there were 11.5% cases of non-scarring alopecia. The most common diagnoses were discoid lupus erythematosus (DLE) (23.1%) and pseudopelade of Brocq (PB) (23.1%), followed by lichen planopilaris (LPP) (15.4%). A significant association was noted between the histological features and the diagnosis, as epidermal atrophy was the most common histological feature in most cases of DLE, followed by periadnexal infiltrates, lymphocytic infiltrates, follicular plugging and basement membrane thickening. In LPP, the most common histological features were perifollicular infiltrates and fibrosis. In PB, the frequently recurring histological features in most cases were the loss of sebaceous glands, dermal fibrosis and epidermal thinning.

Conclusion

In this study, we demonstrated the key role of skin punch biopsy and histology in determining the accurate etiology of scarring alopecia. We found discoid lupus erythematosus and pseudopelade of Brocq to be the most common causes of scarring alopecia, followed by lichen planopilaris.

## Introduction

Alopecia is a dermatological condition which is characterized by the loss of hair from the scalp or body [1]. It is one of the most prevalent globally dermatological conditions and has different etiologies like genetic background, hormonal imbalance, infection or idiopathic causes [2]. Primarily, alopecia is classified as follows: cicatricial (scarring) and non-cicatricial (non-scarring) alopecia [3]. Scarring alopecia is a complex and heterogeneous group of hair disorders which results in scarring of follicles due to hair follicular and sebaceous gland destruction by inflammation, permanent follicular stem cell damage and replacement by connective tissue [4]. Scarring alopecia is further classified on the basis of the predominant inflammatory cell type into lymphocytic that includes discoid lupus erythematosus (DLE), lichen planopilaris (LPP) and pseudopelade of Brocq (PB); neutrophilic that includes folliculitis decalvans (FD), dissecting cellulitis (CD)/folliculitis (perifolliculitis capitis abscedens et suffodiens); and mixed that includes acne keloidalis nuchae (AKN) and erosive pustular dermatosis. Alternatively, non-cicatricial alopecia results due to an alteration in the capillary cycle, which leads to temporary or partial destruction of hair follicles, so hair shedding is followed by hair regrowth [5]. Non-scarring alopecia is further classified into alopecia areata, trichotillomania, traction alopecia, telogen effluvium and androgenic alopecia. The most common type of alopecia is androgenic alopecia among adults [6].

The prevalence and causative etiologies of alopecia have not yet been estimated in Pakistan since there has been little research on alopecia. Owing to different etiologies, the diseases must be accurately diagnosed for appropriate management. The current study determined the clinicopathological features of different types of alopecia in patients undergoing scalp biopsies.

## Materials and methods

We conducted a retrospective cross-sectional study at the Department of Histopathology, Liaquat National Hospital and Medical College, Pakistan. A total of 104 patients were enrolled in the study who underwent scalp biopsy for alopecia over a period of 11 years, from January 2011 to December 2021. Clinicopathological data for all patients diagnosed with alopecia during the study period were recruited. Cases with either missing clinical data or tissue blocks were excluded from the study. Clinical data were obtained from clinical referral forms, which included age, sex of the patients and duration of the lesions. Tissue blocks of the cases were retrieved, and three hematoxylin and eosin-stained tissue sections, along with periodic acid-Schiff (PAS), PAS with diastase, and elastin stains, were examined. PAS Alcian blue and trichrome stains were also done. Key histopathological features, including thickening or thinning of the epidermis, the presence of hyper- or parakeratosis, neutrophilic or lymphocytic exocytosis, flattening of rete ridges, dermoepidermal junction changes, a type of infiltrate in the dermis, perivascular or periadnexal infiltrate, and status of adnexal structures were noted. Dermal fibrosis, mucin and elastin fibers were assessed on special stains, trichrome, PAS Alcian and elastin stains, respectively. Fungal organisms were excluded from PAS with diastase stain. Two histopathologists examined the biopsies, and a histopathological diagnosis was rendered considering the clinical findings.

Written and informed consent was obtained from the patients at the time of skin punch biopsy. All skin punch biopsies were obtained from the lesion site (hairless area) and adjacent area. Biopsies were taken with a 0.3x0.3x0.3 cm bore needle and were preserved in 10% formalin. After formalin fixation, all the skin punch biopsies were bisected, followed by tissue processing and staining as mentioned above. Data analysis was performed using the Statistical Package for Social Sciences (Version 26.0, IBM Inc., Armonk, USA). Chi-square and Fisher’s exact tests were used to check the association. P-values < 0.05 were considered as significant.

## Results

Our study demonstrated that alopecia was more prevalent among females, accounting for 73.1% of cases than males (26.9%). Most of the patients belonged to the age group of <35 years (53.8%), followed by patients between the age of 35 and 50 years (26.9%). The duration of symptoms among most patients was <36 months (69.2%). The type of alopecia in 88.5% of the cases was scarring, while there were 11.5% of cases of non-scarring alopecia. The most common diagnoses were DLE (23.1%) and PB (23.1%), followed by LPP (15.4%), as shown in Table 1.

**Table 1:** Clinicopathological features of population under study SD: standard deviation.

Clinicopathological features	Values
Gender	
Male, n (%)	28 (26.9)
Female, n (%)	76 (73.1)
Age (years)	
Mean±SD	37.54±14.08
Age groups	
≤35 years, n (%)	56 (53.8)
36-50 years, n (%)	28 (26.9)
>50 years, n (%)	20 (19.2)
Duration of symptoms	
Mean±SD	32.12±29.24
Duration of symptoms groups	
≤36 months, n (%)	72 (69.2)
>36 months, n (%)	32 (30.8)
Diagnosis	
Lichen planopilaris, n (%)	16 (15.4)
Discoid lupus erythematosus, n (%)	24 (23.1)
Pseudopelade of Brocq, n (%)	24 (23.1)
Folliculitis decalvans, n (%)	4 (3.8)
Acute suppurative folliculitis, n (%)	4 (3.8)
Pityrosporum folliculitis, n (%)	4 (3.8)
Pityriasis versicolor, n (%)	4 (3.8)
Lupus panniculitis, n (%)	4 (3.8)
Trichotillomania, n (%)	4 (3.8)
Chronic non-specific folliculitis, n (%)	4 (3.8)
Lichen sclerosus, n (%)	4 (3.8)
Nodular prurigo, n (%)	4 (3.8)
Alopecia areata, n (%)	4 (3.8)
Alopecia type	
Scarring, n (%)	92 (88.5)
Non-scarring, n (%)	12 (11.5)

The histological features that showed the highest frequency were lymphocytic infiltrates (42.3%), perivascular infiltrates (38.5%), loss of sebaceous glands (34.6%), perifollicular fibrosis (30.8%) and epidermal atrophy (30.8%), while few of the less common histological features were follicular plugging (26.9%) and perifollicular infiltrates (26.9%), as demonstrated in Table 2.

**Table 2:** Histopathological findings in skin biopsy specimens

Histopathological findings	Values
Epidermal atrophy, n (%)	32 (30.8)
Follicular plugging, n (%)	28 (26.9)
Epidermal thinning, n (%)	20 (19.2)
Flattening of rete ridges, n (%)	16 (15.4)
Acanthosis, n (%)	4 (3.8)
Hyperkeratosis, n (%)	20 (19.2)
Spongiosis, n (%)	4 (3.8)
Subcorneal pustules, n (%)	4 (3.8)
Parakeratosis, n (%)	12 (11.5)
Neutrophilic exocytosis, n (%)	4 (3.8)
Lymphocytic exocytosis, n (%)	4 (3.8)
Vacuolar interface changes, n (%)	20 (19.2)
Basement membrane thickening, n (%)	12 (11.5)
Dermal mucin, n (%)	8 (7.7)
Dermal granulomas, n (%)	4 (3.8)
Neutrophilic infiltrates, n (%)	4 (3.8)
Eosinophilic infiltrates, n (%)	4 (3.8)
Lymphocytic infiltrates, n (%)	44 (42.3)
Histiocytic infiltrates, n (%)	12 (11.5)
Plasmacytic infiltrates, n (%)	12 (11.5)
Perivascular infiltrates, n (%)	40 (38.5)
Periadnexal infiltrates, n (%)	24 (23.1)
Perifollicular infiltrates, n (%)	28 (26.9)
Dermal edema, n (%)	16 (15.4)
Dermal fibrosis, n (%)	20 (19.2)
Loss of sebaceous glands, n (%)	36 (34.6)
Loss of hair follicles, n (%)	16 (15.4)
Perifollicular fibrosis, n (%)	32 (30.8)
Pigment incontinence, n (%)	12 (11.5)
Loss of elastic fibers, n (%)	8 (7.7)

Table 3 shows the association of disease with gender, age and duration of symptoms. PB and DLE showed female predominance, with most patients belonging to the age group of <35 years of age. Similarly, LPP mostly affected females, whereas the common age group of patients was above 50 years. FD, pityrosporum folliculitis, lupus panniculitis, lichen sclerosus, alopecia areata and some of the less common diseases also showed female predominance, whereas acute suppurative folliculitis, pityriasis versicolor, trichotillomania and nodular prurigo showed male predominance. Our study showed that the duration of symptoms in most cases of DLE, PB, FD, pityrosporum folliculitis, lupus panniculitis, trichotillomania, lichen sclerosus, nodular prurigo and chronic non-specific folliculitis was <36 months, while the duration of symptoms in cases of LPP, acute suppurative folliculitis, pityriasis versicolor and alopecia areata in most cases was >36 months. Scarring alopecia was more common among females (78.3%) with a duration of symptoms of <36 months (73.9%), whereas non-scarring alopecia was more prevalent among men (66.7%) with a duration of symptoms of >36 months (66.7%).

**Table 3:** Association of clinical features with the diagnosis *p-value significant as <0.05.

Diagnosis	Values						
	Gender		Age (years)			Duration of symptoms	
	Male	Female	≤35 years	36-50 years	>50 years	≤36 months	>36 months
Lichen planopilaris, n (%)	4 (25)	12 (75)	4 (25)	4 (25)	8 (50)	4 (25)	12 (75)
Discoid lupus erythematosus, n (%)	4 (16.7)	20 (83.3)	16 (66.7)	8 (33.3)	0 (0)	20 (83.3)	4 (16.7)
Pseudopelade of Brocq, n (%)	0 (0)	24 (100)	12 (50)	4 (16.7)	8 (33.3)	20 (83.3)	4 (16.7)
Folliculitis decalvans, n (%)	0 (0)	4 (100)	4 (100)	0 (0)	0 (0)	4 (100)	0 (0)
Acute suppurative folliculitis, n (%)	4 (100)	0 (0)	4( 100)	0 (0)	0 (0)	0 (0)	4 (100)
Pityrosporum folliculitis, n (%)	0 (0)	4 (100)	0 (0)	4 (100)	0 (0)	4 (100)	0 (0)
Pityriasis versicolor, n (%)	4 (100)	0 (0)	4 (100)	0 (0)	0 (0)	0 (0)	4 (100)
Lupus panniculitis, n (%)	0 (0)	4 (100)	4 (100)	0 (0)	0 (0)	4 (100)	0 (0)
Trichotillomania, n (%)	4 (100)	0 (0)	4 (100)	0 (0)	0 (0)	4 (100)	0 (0)
Chronic non-specific folliculitis, n (%)	4 (100)	0 (0)	4 (100)	0 (0)	0 (0)	4 (100)	0 (0)
Lichen sclerosus, n (%)	0 (0)	4 (100)	0 (0)	4 (100)	0 (0)	4 (100)	0 (0)
Nodular prurigo, n (%)	4 (100)	0 (0)	0 (0)	0 (0)	4 (100)	4 (100)	0 (0)
Alopecia areata, n (%)	0 (0)	4 (100)	0 (0)	4 (100)	0 (0)	0 (0)	4 (100)
	p-value<0.001*		p-value<0.001*			p-value<0.001*	
Alopecia type							
Scarring, n (%)	20 (21.7)	72 (78.3)	48 (52.2)	24 (26.1)	20 (21.7)	68 (73.9)	24 (26.1)
Non-scarring, n (%)	8 (66.7)	4 (33.3)	8 (66.7)	4 (33.3)	0 (0)	4 (33.3)	8 (66.7)
	p-value<0.05*		p-value=0.203			p-value<0.05*	

Table 4 shows the association between histological features and diagnosis. A significant association was noted between the histological features and the diagnosis, as epidermal atrophy was the most common histological feature in most cases of DLE, followed by periadnexal infiltrates, lymphocytic infiltrates, follicular plugging and basement membrane thickening. In LPP, the most common histological features were perifollicular infiltrates and fibrosis. In PB, the frequently recurring histological features in most cases were the loss of sebaceous glands, dermal fibrosis and epidermal thinning. Other common features were epidermal atrophy, flattening of rete ridges, vacuolar interface changes and perifollicular fibrosis.

**Table 4:** Association of histopathological findings with the diagnosis *p-value significant as <0.05.

Histopathological findings	Values													p-value
	Lichen planopilaris	Discoid lupus erythematosus	Pseudopelade of Brocq	Folliculitis decalvans	Acute suppurative folliculitis	Pityrosporum folliculitis	Pityriasis versicolor	Lupus panniculitis	Trichotillomania	Chronic non-specific folliculitis	Lichen sclerosus	Nodular prurigo	Alopecia areata	
Epidermal atrophy, n (%)	0 (0)	24 (75)	8 (25)	0 (0)	0 (0)	0 (0)	0 (0)	0 (0)	0 (0)	0 (0)	0 (0)	0 (0)	0 (0)	<0.001*
Follicular plugging, n (%)	0 (0)	12 (42.9)	4 (14.3)	0 (0)	0 (0)	4 (14.3)	0 (0)	4 (14.3)	4 (14.3)	0 (0)	0 (0)	0 (0)	0 (0)	<0.001*
Epidermal thinning, n (%)	0 (0)	0 (0)	12 (60)	0 (0)	0 (0)	0 (0)	0 (0)	4 (20)	0 (0)	0 (0)	4 (20)	0 (0)	0 (0)	<0.001*
Flattening of rete ridges, n (%)	0 (0)	8 (50)	8 (50)	0 (0)	0 (0)	0 (0)	0 (0)	0 (0)	0 (0)	0 (0)	0 (0)	0 (0)	0 (0)	0.12
Acanthosis, n (%)	4 (100)	0 (0)	0 (0)	0 (0)	0 (0)	0 (0)	0 (0)	0 (0)	0 (0)	0 (0)	0 (0)	0 (0)	0 (0)	0.17
Hyperkeratosis, n (%)	4 (20)	0 (0)	4 (20)	0 (0)	0 (0)	0 (0)	0 (0)	0 (0)	4 (20)	0 (0)	4 (20)	0 (0)	0 (0)	<0.001*
Spongiosis, n (%)	0 (0)	0 (0)	0 (0)	0 (0)	4 (100)	0 (0)	0 (0)	0 (0)	0 (0)	0 (0)	0 (0)	0 (0)	0 (0)	<0.001*
Subcorneal pustules, n (%)	0 (0)	0 (0)	0 (0)	0 (0)	4 (100)	0 (0)	0 (0)	0 (0)	0 (0)	0 (0)	0 (0)	0 (0)	0 (0)	<0.001*
Parakeratosis, n (%)	0 (0)	4 (33.3)	0 (0)	0 (0)	0 (0)	0 (0)	0 (0)	0 (0)	4 (33.3)	0 (0)	0 (0)	4 (33.3)	0 (0)	<0.001*
Neutrophilic exocytosis, n (%)	0 (0)	0 (0)	0 (0)	0 (0)	4 (100)	0 (0)	0 (0)	0 (0)	0 (0)	0 (0)	0 (0)	0 (0)	0 (0)	<0.001*
Lymphocytic exocytosis, n (%)	0 (0)	0 (0)	0 (0)	0 (0)	0 (0)	0 (0)	0 (0)	0 (0)	0 (0)	0 (0)	4 (100)	0 (0)	0 (0)	<0.001*
Vacuolar interface changes, n (%)	4 (20)	8 (40)	8 (40)	0 (0)	0 (0)	0 (0)	0 (0)	0 (0)	0 (0)	0 (0)	0 (0)	0 (0)	0 (0)	<0.001*
Basement membrane thickening, n (%)	0 (0)	12 (100)	0 (0)	0 (0)	0 (0)	0 (0)	0 (0)	0 (0)	0 (0)	0 (0)	0 (0)	0 (0)	0 (0)	<0.001*
Dermal mucin, n (%)	0 (0)	8 (100)	0 (0)	0 (0)	0 (0)	0 (0)	0 (0)	0 (0)	0 (0)	0 (0)	0 (0)	0 (0)	0 (0)	<0.001*
Dermal granulomas, n (%)	0 (0)	0 (0)	0 (0)	0 (0)	0 (0)	0 (0)	0 (0)	0 (0)	0 (0)	4 (100)	0 (0)	0 (0)	0 (0)	<0.001*
Neutrophilic infiltrates, n (%)	0 (0)	0 (0)	0 (0)	0 (0)	0 (0)	0 (0)	0 (0)	0 (0)	0 (0)	0 (0)	4 (100)	0 (0)	0 (0)	<0.001*
Eosinophilic infiltrates, n (%)	4 (100)	0 (0)	0 (0)	0 (0)	0 (0)	0 (0)	0 (0)	0 (0)	0 (0)	0 (0)	0 (0)	0 (0)	0 (0)	<0.001*
Lymphocytic Infiltrates, n (%)	8 (18.2)	16 (36.4)	4 (9.1)	4 (9.1)	0 (0)	0 (0)	0 (0)	4 (9.1)	0 (0)	0 (0)	0 (0)	4 (9.1)	4 (9.1)	<0.001*
Histiocytic infiltrates, n (%)	0 (0)	4 (33.3)	0 (0)	4 (33.3)	0 (0)	0 (0)	0 (0)	0 (0)	0 (0)	0 (0)	0 (0)	4 (33.3)	0 (0)	<0.001*
Plasmacytic Infiltrates, n (%)	0 (0)	4 (33.3)	0 (0)	4 (33.3)	0 (0)	0 (0)	0 (0)	4 (33.3)	0 (0)	0 (0)	0 (0)	0 (0)	0 (0)	<0.001*
Perivascular infiltrates, n (%)	4 (10)	12 (30)	4 (10)	0 (0)	4 (10)	0 (0)	4 (10)	4 (10)	0 (0)	0 (0)	0 (0)	4 (10)	4 (10)	<0.001*
Periadnexal infiltrates, n (%)	0 (0)	16 (66.7)	0 (0)	0 (0)	4 (16.7)	0 (0)	0 (0)	4 (16.7)	0 (0)	0 (0)	0 (0)	0 (0)	0 (0)	<0.001*
Perifollicular infiltrates, n (%)	12 (42.9)	8 (28.6)	0 (0)	0 (0)	0 (0)	4 (14.3)	4 (14.3)	0 (0)	0 (0)	0 (0)	0 (0)	0 (0)	0 (0)	<0.001
Dermal edema, n (%)	0 (0)	8 (50)	0 (0)	0 (0)	0 (0)	0 (0)	0 (0)	4 (25)	0 (0)	0 (0)	4 (25)	0 (0)	0 (0)	<0.001*
Dermal fibrosis, n (%)	0 (0)	0 (0)	12 (60)	4 (20)	0 (0)	0 (0)	0 (0)	0 (0)	0 (0)	0 (0)	0 (0)	4 (25)	0 (0)	<0.001*
Loss of sebaceous glands, n (%)	8 (22.2)	4 (11.1)	20 (55.6)	4 (11.1)	0 (0)	0 (0)	0 (0)	0 (0)	0 (0)	0 (0)	0 (0)	0 (0)	0 (0)	<0.001
Loss of hair follicles, n (%)	8 (50)	0 (0)	4 (25)	0 (0)	0 (0)	0 (0)	0 (0)	0 (0)	0 (0)	0 (0)	0 (0)	0 (0)	4 (25)	<0.001*
Perifollicular fibrosis, n (%)	12 (37.5)	4 (12.5)	8 (25)	4 (12.5)	0 (0)	0 (0)	0 (0)	0 (0)	0 (0)	0 (0)	0 (0)	0 (0)	4 (12.5)	<0.001*
Pigment incontinence, n (%)	4 (33.3)	4 (33.3)	4(33.3)	0 (0)	0 (0)	0 (0)	0 (0)	0 (0)	0 (0)	0 (0)	0 (0)	0 (0)	0 (0)	0.99
Loss of elastic fibers, n (%)	8 (100)	0 (0)	0(0)	0 (0)	0 (0)	0 (0)	0 (0)	0 (0)	0 (0)	0 (0)	0 (0)	0 (0)	0 (0)	<0.001*
Alopecia type														
Scarring, n (%)	16 (17.4)	24 (26.1)	24 (26.1)	4 (4.3)	4 (4.3)	4 (4.3)	0 (0)	4 (4.3)	0 (0)	4 (4.3)	4 (4.3)	4 (4.3)	0 (0)	<0.001*
Non-scarring, n (%)	0 (0)	0 (0)	0(0)	0 (0)	0 (0)	0 (0)	4 (33.3)	0 (0)	4 (33.3)	0 (0)	0 (0)	0 (0)	4 (33.3)	

## Discussion

In this study, we found that scarring alopecia was more commonly biopsied. Additionally, it was also noted that DLE and PB were the most frequent causes of scarring alopecia in our study population, followed by LPP. A study was conducted by Madubuko and Okwara in Southern Nigeria; they observed 106 patients with alopecia and, contrary to our study, concluded that males were more affected by alopecia, accounting for 61.3% of the cases than females constituting 38.7% of the cases [7]. Alternatively, in another study conducted in Taiwan at National Cheng Kung University Hospital between 1988 and 2016, 89 patients with scarring alopecia were observed and they found that the most frequent type of alopecia was DC (30.3%), followed by LPP (23.5%), central centrifugal cicatricial alopecia (CCCA) (12.4%) and AKN (12.4%). They found FD, DLE and PB among the less common types. Similar to our study, they noted female predominance in DLE, LPP and PB, while DC and AKN showed male predominance [8]. Inchara et al. studied 37 cases of scarring alopecia and concluded that lupus erythematosus was the most common cause of scarring alopecia among Indian patients (49%), followed by LPP (41%), folliculitis and alopecia areata [9]. They found that epidermal atrophy, papillary dermal fibrosis and mucin and peribulbar inflammation were the most common histological features in their patients with DLE, and the common histological features of LPP were normal epidermis and peri-infundibular infiltrate [9]. In our study, we found that the common histological features of DLE among our patients were epidermal atrophy, periadnexal infiltrates, follicular plugging and basement membrane thickening and that in LPP, the most common histological feature in most cases was perifollicular infiltrate.

In another study conducted in India, 40 patients with cicatricial alopecia were recruited; they found that the cause of alopecia in 27.5% of the cases was LPP, being the most common diagnosis, followed by DLE in 25% of the cases, and 20% patients had PB. Similar to our study, they found female predominance, while most of the patients fell into the age group ranging from 41 to 50 years [10]. Another retrospective study conducted in India found that DLE was the most frequent cause of primary cicatricial alopecia (PCA), the second most common cause being LPP in 20.7% of the patients; the other causes were 12.5% of DC and 8.3% each PB, FD and frontal fibrosing alopecia. They found males to be more frequently affected; the common histological features of DLE that they observed were follicular plugging, superficial and deep perifollicular and perivascular lymphocytic infiltrate, vacuolar changes in the basal layer and necrotic keratinocytes [11].

In another retrospective study conducted at the University of British Columbia Hair Clinic, Vancouver, Canada, 112 patients with PCA over a five-year period were studied, representing only 3.2% of all trichologic cases; similar to our study, they found most cases were histopathologically of lymphocytic infiltrate type of PCA (4:1), among which the most common sub-types were DLE (33.9%), followed by PB (24.1%) and LPP (22.3%) [12]. A study was conducted in China on 59 patients with PCA; they found that the ratio of neutrophilic to lymphocytic cicatricial alopecias was about 1.3:1 in this group. The most frequent disorder was FD (40.7%), followed by DLE (22.0%), PB (15.3%) and DC (15.3%); they found that LPP and alopecia mucinosa were among the less common types [13]. Chiramel et al. conducted a study in North India: 120 patients with alopecia observed; they found that 90% of the patients had non-cicatricial alopecia, while the rest of the patients (30%) had cicatricial alopecia; their study also showed female predominance, and the mean age of patients was 24.9 years; and they found that alopecia areata was the most common diagnosis [14]. Kumar and Yelikar studied 32 cases of scarring alopecia and concluded that the lymphocytic type of scarring alopecia was the most common, with lupus erythematosus (79.16%) being the most common cause [15].

Discoid lupus erythematosus (DLE)

Our study showed that DLE was more common among females of <35 years of age, and the duration of symptoms was <36 months. The most common histological features that we observed in our patients with DLE were epidermal atrophy, periadnexal infiltrates, lymphocytic infiltrates, histoplasmacytic infiltrates, follicular plugging and basement membrane thickening, whereas the less common features were loss of sebaceous glands and hair follicles, flattening of rete ridges, dermal edema, dermal mucin, vacuolar interface changes, perifollicular infiltrates and fibrosis and pigment incontinence. Cummins et al. studied the key histological features of scarring alopecia and found that histologically DLE is characterized by follicular hyperkeratosis, vacuolar degeneration of basal keratinocytes, basement membrane thickening, destruction of sebaceous glands and periadnexal and perivascular lymphoplasmacytic inflammatory infiltrates in both deep and superficial dermis [16].

Lichen planopilaris (LPP)

In our study, LPP showed female predominance, belonging to the adult age group of >50 years with a duration of symptoms of >36 months. The biopsy results of the patients with LPP in our study showed lymphocytic perifollicular infiltrates, perifollicular fibrosis, loss of hair follicles and sebaceous glands and increased elastic fibers in the dermis. Tandon et al. studied the histology of LPP in 27 patients and concluded that the loss of arrector pili muscle and sebaceous glands, perifollicular and perivascular lymphocytic infiltrates, perifollicular mucinous fibroplasia and interfollicular mucin loss in the upper dermis were the most common features [17].

Pseudopelade of Brocq (PB)

In our study, we concluded that PB was more common among females of <35 years of age and with a duration of symptoms of <36 months, the frequently recurring histological characteristics in cases of PB were loss of sebaceous gland, dermal fibrosis, epidermal thinning, epidermal atrophy, flattening of rete ridges and perifollicular fibrosis. A study was conducted in India, in which they studied clinicopathological features of PCA and found that PB was more common among females, and the histological characteristics that they observed among their patients were lymphocytic Infiltrates around perifollicular areas associated with the absence of rete ridges with epidermis being normal or atrophic. They also observed the loss of sebaceous glands and hair follicles, while the later stages of disease demonstrated fibrosis and absence of inflammatory infiltrates [18].

Limitations

The limitations of our study included single institution data, failure to observe treatment response and lack of follow-up of patients. Therefore, it is recommended that multi-center studies should be conducted to determine the relative frequencies of alopecia in our population and their histological characteristics. Moreover, direct immunofluorescence (DIF) is one of the important diagnostic tools in the evaluation of some dermatological diagnoses, especially DLE, in the context of scarring alopecia, which was not performed in our study.

## Conclusions

Although non-scarring alopecia is more prevalent, the clinical diagnosis of scarring alopecia is challenging, and therefore, scarring alopecia is more commonly biopsied than non-scarring alopecia, as seen in our study. In this study, we found DLE and PB, followed by LPP to be the most common causes of scarring alopecia in our population. Our study also demonstrated the common histological features of different types of alopecia and emphasized the crucial role of skin punch biopsy and histopathology for the accurate diagnosis of the disease and its treatment.
